# Supplementary material for: Integrated analysis of lymphocyte infiltration-associated lncRNA for ovarian cancer via TCGA, GTEx and GEO datasets
Source: PeerJ. 2020 May 7;8:e8961. doi: 10.7717/peerj.8961 (PMC7211406; doi:10.7717/peerj.8961)
Supplement: Supplemental Information 1 [file peerj-08-8961-s001.docx]

**
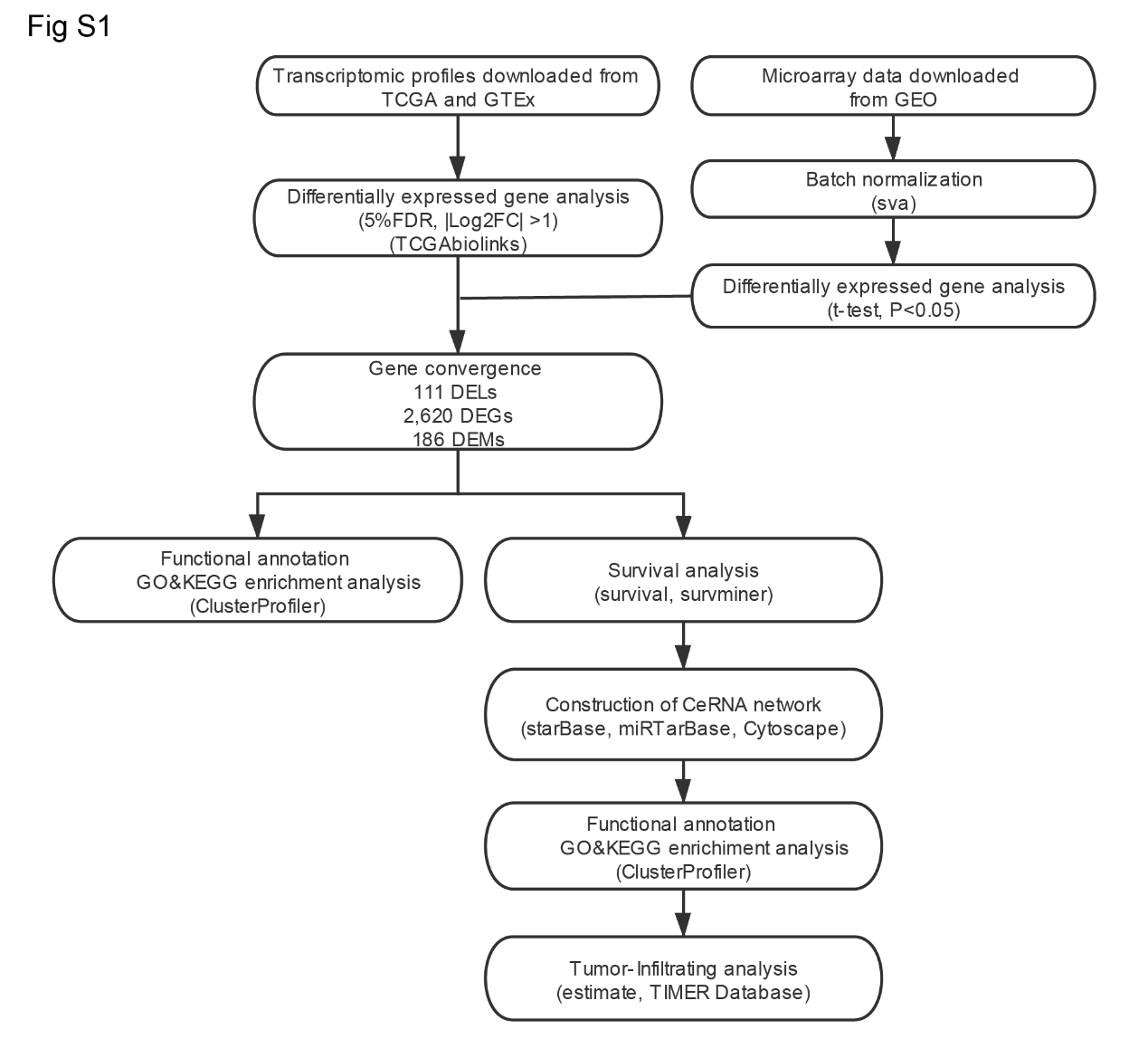
**

Figure S1 Flowchart for bioinformatics analysis of datasets from TCGA, GTEx and GEO.


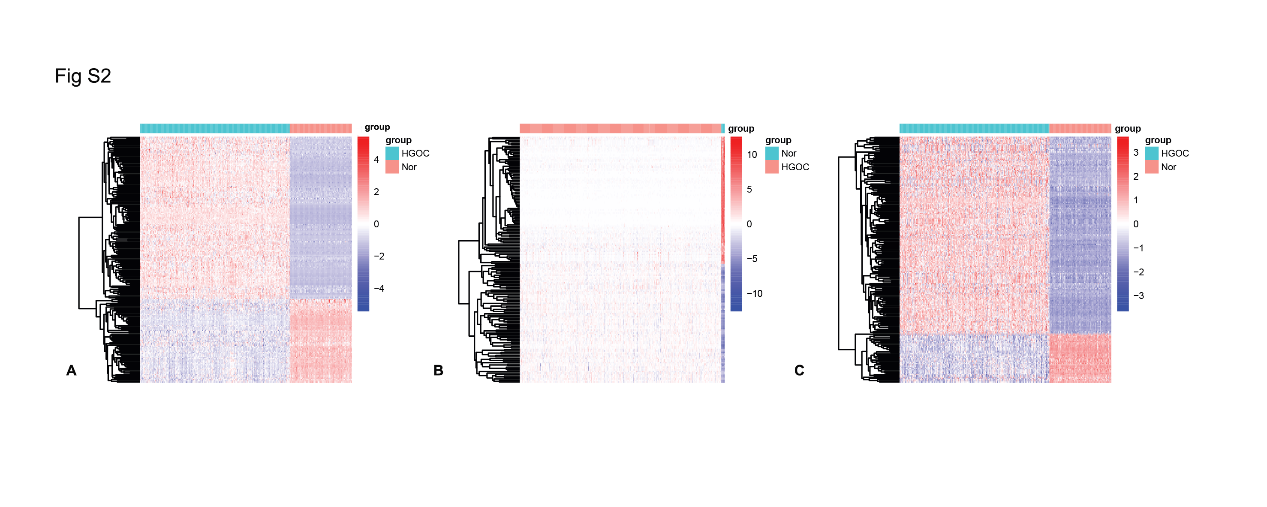


Figure S2 Heat map analysis of differentially expressed lncRNAs (A), miRNAs (B), and genes (C). The horizontal axis denoted the cluster analysis of each sample. The longitudinal axis indicated the cluster analysis of lncRNA, miRNA, or gene. High- or low-relative expression is displayed as a red or blue strip, respectively.


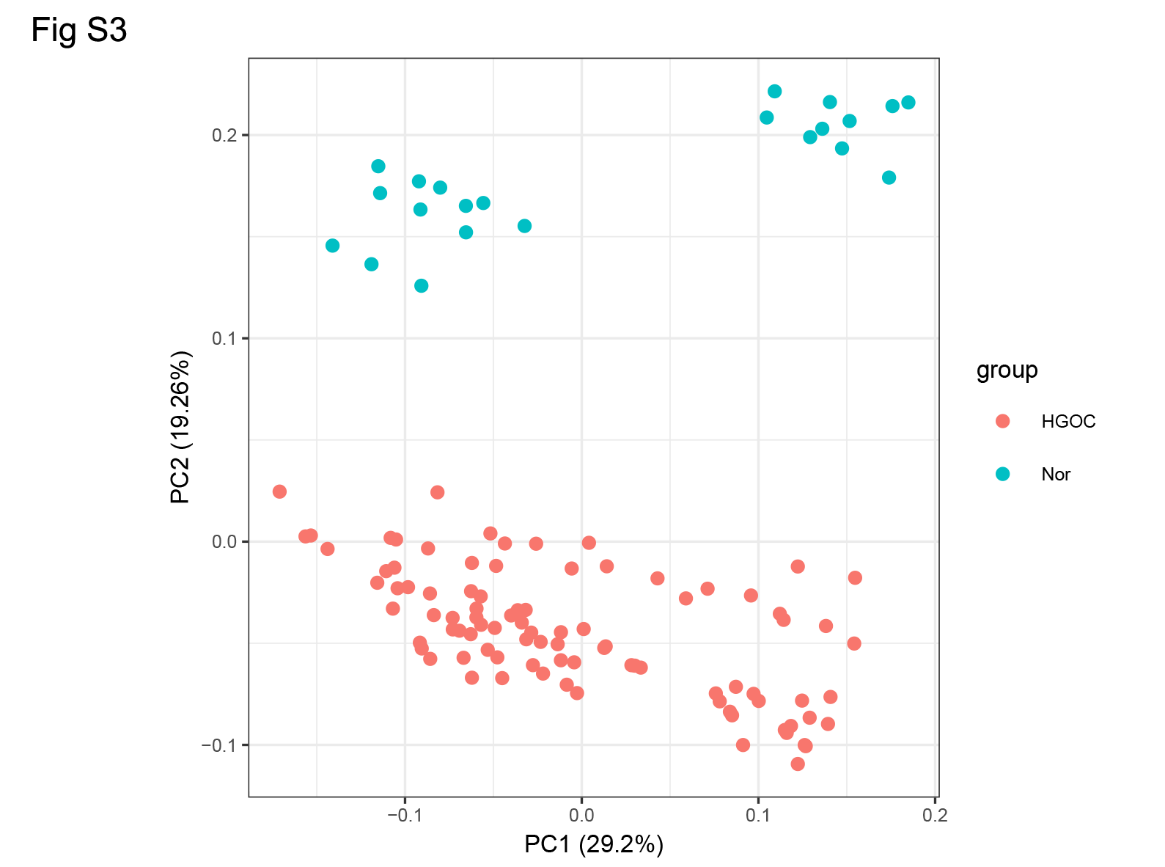


Figure S3 Results from the principal component analysis for integrated microarray studies downloaded from the GEO dataset. Two-dimensional plots of normal and tumor groups with the top two principal components. Horizontal and vertical axes represent the distribution of each sample within PCA1 and PCA2 respectively. PCA1: principle component 1; PCA2: principal component 2.


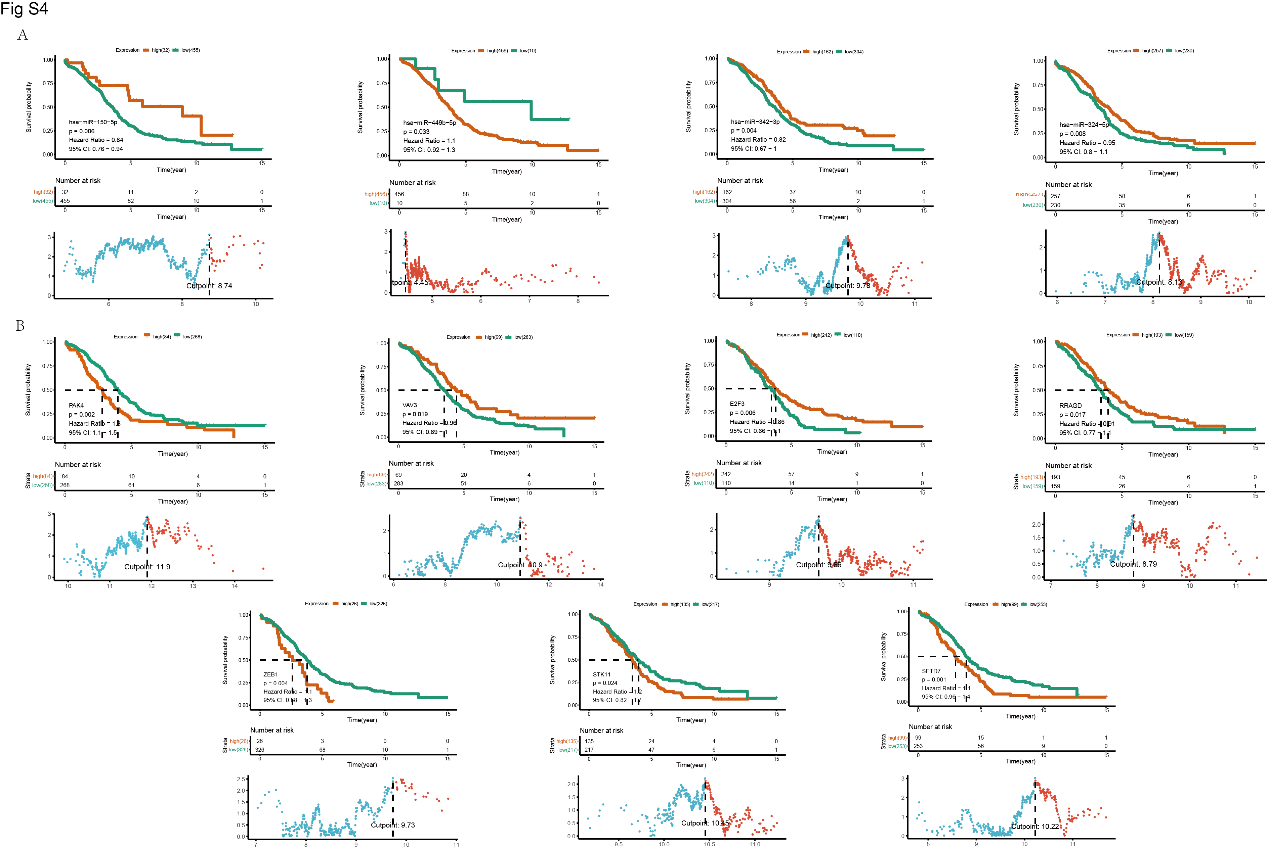


Figure S4 Survival analysis of miRNAs and mRNAs in ceRNA network.

Kaplan–Meier analysis of differentially expressed miRNAs(A), and mRNAs(B) in ceRNA network by comparing the higher(red) and lower(green) expressions with overall survival outcomes for patients with HGSOC. P-value set for this analysis is less than 0.05.

The bottom part shows the threshold set by R package. Horizontal axis: the expression level of a certain gene, Vertical axis: standardized Log-Rank statistic. Dotted line: the cutoff value for gene expression.

**
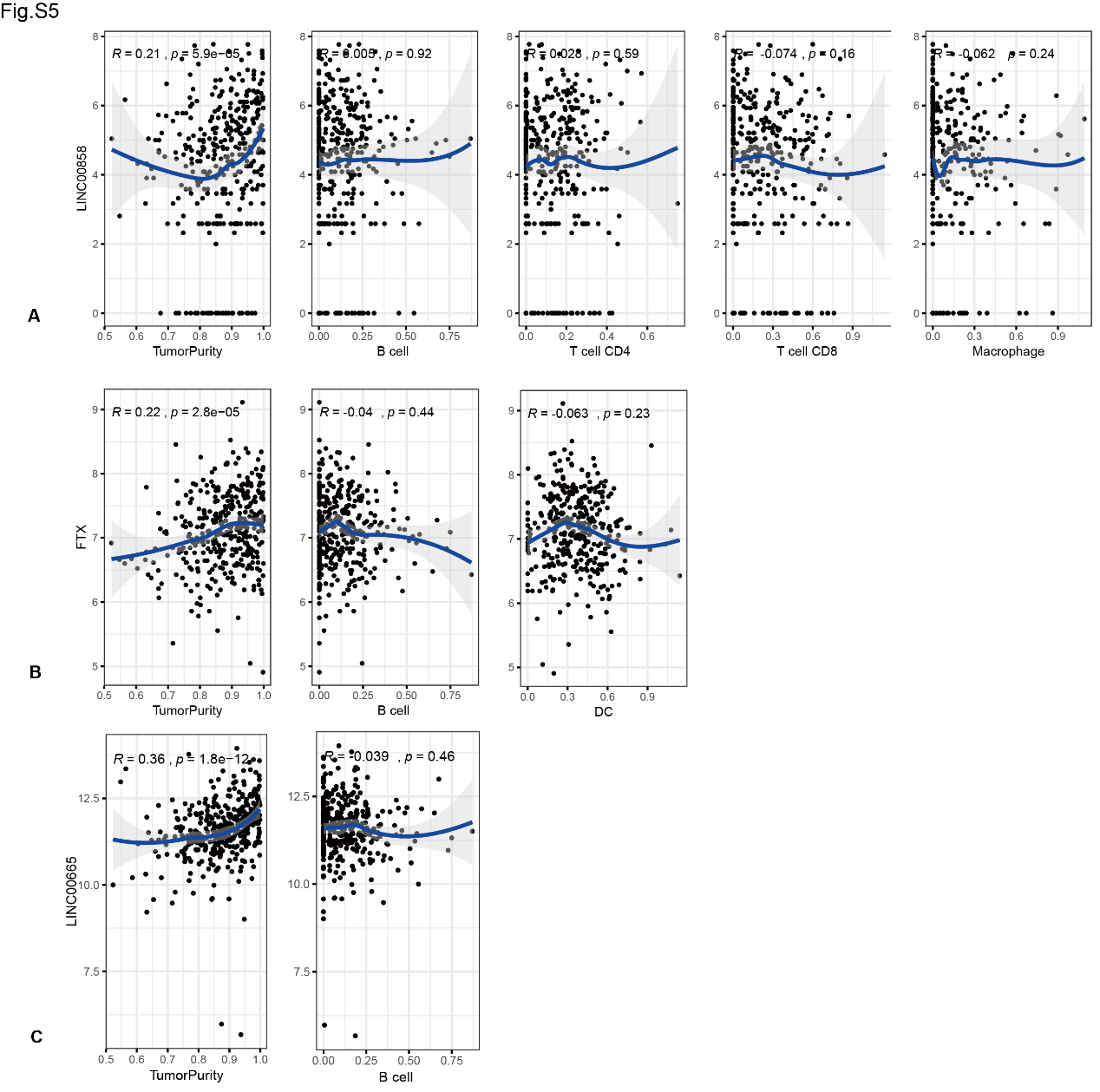
**

Figure S5 Correlation of lncRNAs, LINC00858 (A), FTX (B), and LINC00665 (C), expression with inﬁltrating levels of tumor purity (first column) and lymphocyte infiltration level in high-grade serous ovarian cancer.

Table S5 KEGG pathway enrichment for differentially expressed genes

| ID | Description | P value | Count | Regulation |
| --- | --- | --- | --- | --- |
| hsa04110 | Cell cycle | 8.01^E-05^ | 35 | upregulation |
| hsa03030 | DNA replication | 0.00181 | 12 | upregulation |
| hsa03010 | Ribosome | 0.002645 | 15 | upregulation |
| hsa01230 | Biosynthesis of amino acids | 0.015409 | 14 | upregulation |
| hsa00190 | Oxidative phosphorylation | 0.023202 | 13 | upregulation |
| hsa03440 | Homologous recombination | 0.026669 | 10 | upregulation |
| hsa04060 | Cytokine-cytokine receptor interaction | 0.034683 | 29 | upregulation |
| hsa04218 | Cellular senescence | 0.035041 | 27 | upregulation |
| hsa04657 | IL-17 signaling pathway | 0.049274 | 11 | upregulation |
| hsa04022 | cGMP-PKG signaling pathway | 5.29^E-05^ | 17 | downregulation |
| hsa04610 | Complement and coagulation cascades | 0.000681 | 12 | downregulation |
| hsa04072 | Phospholipase D signaling pathway | 0.014802 | 16 | downregulation |
| hsa04350 | TGF-beta signaling pathway | 0.021213 | 11 | downregulation |
| hsa04140 | Autophagy - animal | 0.023443 | 13 | downregulation |
| hsa04014 | Ras signaling pathway | 0.032845 | 21 | downregulation |
| hsa04371 | Apelin signaling pathway | 0.038948 | 14 | downregulation |
| hsa04630 | JAK-STAT signaling pathway | 0.038948 | 14 | downregulation |

Table S6 GO biological process for differentially expressed genes

| ID | Description | Count | P value | Regulation |
| --- | --- | --- | --- | --- |
| GO:0048285 | organelle fission | 93 | 5.12^E-15^ | upregulation |
| GO:0007059 | chromosome segregation | 91 | 9.76^E-21^ | upregulation |
| GO:0000280 | nuclear division | 90 | 2.27^E-16^ | upregulation |
| GO:0098813 | nuclear chromosome segregation | 81 | 1.26^E-19^ | upregulation |
| GO:0071103 | DNA conformation change | 73 | 2.27^E-16^ | upregulation |
| GO:0000819 | sister chromatid segregation | 70 | 1.26^E-19^ | upregulation |
| GO:0140014 | mitotic nuclear division | 68 | 1.51^E-15^ | upregulation |
| GO:1901987 | regulation of cell cycle phase transition | 62 | 0.000873164 | upregulation |
| GO:0006260 | DNA replication | 59 | 6.85^E-09^ | upregulation |
| GO:0006323 | DNA packaging | 56 | 5.99^E-14^ | upregulation |
| GO:0045787 | positive regulation of cell cycle | 56 | 0.000233921 | upregulation |
| GO:0051052 | regulation of DNA metabolic process | 56 | 0.000976283 | upregulation |
| GO:1901990 | regulation of mitotic cell cycle phase transition | 55 | 0.005152821 | upregulation |
| GO:0048568 | embryonic organ development | 55 | 0.008049245 | upregulation |
| GO:0009314 | response to radiation | 54 | 0.025804851 | upregulation |
| GO:1901653 | cellular response to peptide | 38 | 0.029874989 | downregulation |
| GO:0019216 | regulation of lipid metabolic process | 38 | 0.042661545 | downregulation |
| GO:0007178 | transmembrane receptor protein serine/threonine kinase signaling pathway | 36 | 0.042661545 | downregulation |
| GO:0071375 | cellular response to peptide hormone stimulus | 34 | 0.042661545 | downregulation |
| GO:0071383 | cellular response to steroid hormone stimulus | 33 | 0.01133506 | downregulation |
| GO:0061448 | connective tissue development | 29 | 0.029528939 | downregulation |
| GO:0009755 | hormone-mediated signaling pathway | 27 | 0.047752345 | downregulation |
| GO:0043401 | steroid hormone mediated signaling pathway | 26 | 0.014684241 | downregulation |
| GO:0010517 | regulation of phospholipase activity | 11 | 0.049805782 | downregulation |
| GO:0097035 | regulation of membrane lipid distribution | 9 | 0.044563787 | downregulation |
| GO:0030318 | melanocyte differentiation | 8 | 0.014684241 | downregulation |
| GO:0045332 | phospholipid translocation | 8 | 0.014684241 | downregulation |
| GO:0034204 | lipid translocation | 8 | 0.016669149 | downregulation |
| GO:0048070 | regulation of developmental pigmentation | 6 | 0.042661545 | downregulation |
| GO:0032429 | regulation of phospholipase A2 activity | 5 | 0.035988155 | downregulation |

Table S7 OS-associated differentially expressed genes

| Gene type | Regulation | Gene name |
| --- | --- | --- |
| DEG | UP | CLDN6 HTR3A FOLR1 CLDN16 SLC34A2 MMP7 SCGB2A1 SOX11 CRABP1 CAPN13 DPEP3 C6orf223 VTCN1 PHOX2A CDKN2A CXCL17 XAGE2 PTH2R NXPH4 KLK5 FAM181A SMPDL3B ILDR1 AOC1 SMIM22 TMPRSS3 KRT7 LIN28B AQP5 EPCAM PRSS21 CLDN9 GJB1 LCN2 ROS1 GPR12 LAMP3 HIST1H3B FOXA2 NOTUM SCGB1D2 TMPRSS4 GLYATL2 FOXJ1 HOXB8 CACNG4 CD24 FOXQ1 XDH GLDC KRT23 MAL2 SST AP1M2 PBK MMP1 PADI3 ETV4 PKHD1 C1orf210 GMNC CDC20 GPR39 IGFL1 PRSS8 E2F8 ESRP1 LRRC55 CYP24A1 UCP2 INAVA CRABP2 KCNS1 FABP6 LYPD6B SPDEF KDF1 RAB25 NEK2 TACSTD2 EHF ELF3 GRHL2 PDE6G PCLAF C1orf116 CEP55 SYCE3 HIST1H3C LGSN CKAP2L NEIL3 BIRC5 SKA3 TTK EPHX4 DGKK BIK SP8 GPR19 ST14 MISP CCNA1 RDM1 CP MELK C2orf54 MYH14 PPP1R14D GJA8 EDN2 SLC35D3 PRSS22 KLHL14 FAM83H BSPRY ACY3 AQP6 METTL7B RRM2 PKP3 LAD1 CYP2F1 HMGA1 C6orf132 RIPK4 CCL20 MAJIN NECTIN4 FRMPD1 RASAL1 SAPCD2 UGT8 CCL25 APELA SERINC2 LGALS14 ADAMDEC1 CDH18 ASF1B FOXN1 KLHDC7B SHISA9 MACC1 CDH6 GAL3ST3 SGO1 FAM83E HES2 DIRAS2 POU2F3 SPP1 SLAMF9 TK1 GJB3 FAM111B CLDN19 C15orf48 CDH12 DCDC2 KIF4A SPINT1 E2F2 OASL TFAP2A CHST6 VPREB3 GSDMC LRRC15 TMC4 ZIC3 HIST1H2AJ BCL2L14 PYY UBE2T GABRP S100A14 CRB3 CCNE1 LRRC8E HOXD1 HRK SCNN1A TFAP2C DYDC2 NCAPH RIPPLY3 PPP1R14C SKAP1 S100A2 CYP1A2 TGM7 DLX4 NPW KRT86 NRTN CLSPN SLCO6A1 OVOL1 ATP10B ESCO2 CCL7 SLC4A11 IFNL1 ESPN SMKR1 DTL FUT3 CCNB2 FOXE3 HOXB4 VSTM2L CDC45 TMPRSS13 GALP NLRP7 GRHL3 ECT2 ASRGL1 CRTAC1 EXO1 ERFE IFI27 PLS1 COMP ASCL2 CELSR1 HMGA2 KIF18A PRRG2 HAPLN1 EVA1A ERBB3 DNAAF3 TTC39A ASPM LMX1B IL4I1 DRC1 SLITRK1 HIST1H2AL CKS2 PKMYT1 SPX LPAR3 KIF1A CDCA5 BICDL2 NEURL3 DAND5 HIST1H2BH CDK1 ESPL1 PRR7 CENPA ATP1A3 CENPF RUNX3 PADI2 CACNA1B TPD52 CDKN3 VAV3 ISG15 BFSP2 SUCNR1 TBX20 ARL4C CENPM OAS2 P2RY6 NDC80 CLDN10 HIST1H2AB RAD51AP1 CCR3 CELSR2 CYP4F11 PQLC2L PAQR4 CCL17 ZNF560 C2orf88 GABRQ CAMK2N2 TTC22 MIOX CHRNA9 LEMD1 BLM ROPN1L TMEM61 HSH2D KPNA2 POLQ FAT2 MECOM SBK1 F12 CHODL LMX1A ELF5 SERPINB10 RSPH6A ELOVL3 SPTBN2 LMO1 TLCD1 HIST1H2BB TMEM45B ST6GALNAC2 NUP210 VNN3 ARHGAP11A KCNK15 NOG PROM2 CCNB1 CDCA7 PRAME ZWINT RPL39L PLAC1 DLEU7 GALNT6 CCDC187 SRD5A3 CHRNA6 POC1A PHOSPHO1 CA2 HS3ST4 CXCL13 TRIP13 RSPO4 MAD2L1 PLK1 L1CAM MEOX1 SPON1 RCAN3 MAPK15 PCP2 LAPTM4B ADTRP MFAP5 SH2D4B ABHD11 HMGB3 SLAMF7 SLC27A2 HIST1H3A BAIAP2L1 RAB39B PRKCG CENPK KCNN4 SLC2A1 OR1Q1 CD3G IGFBPL1 KCNJ16 UNC93B1 CSMD2 MPZL2 FGF8 TNFRSF12A ONECUT3 CCDC167 THEM6 CCND1 FAM155B KAZALD1 TACC3 SYNE4 XPR1 DLGAP3 USP18 KRTCAP3 SULT1C2 ACRV1 HUNK IL32 PMCH ALPK2 GRHL1 C3orf80 P2RY2 PITX3 BTLA APOA1 C1orf87 SEMA3F FGF23 LARGE2 CXCR4 VWDE SHCBP1 TEKT2 ESM1 TNFAIP6 SEMA5B RHPN1 CKS1B ITGB8 BARX1 TRPV4 KCNQ3 NCEH1 GALNT3 MZB1 OCIAD2 ZBTB42 ZDHHC12 IL21R DLL3 MARVELD3 RAB11FIP4 SYTL1 ABCA4 SLC6A12 MREG TRIB1 DHCR24 IDH2 MUC4 NDUFB9 JPT1 NKAIN1 SHISA8 BACE2 HPDL CATSPER1 PPP1R14B PRR18 VANGL1 C2orf15 GNG13 EXOSC4 ANKFN1 FAM178B GPT2 CLEC5A MPP7 EXPH5 LACTB2 TDRKH LRRC8D CD207 MPZL3 C9orf16 PSENEN ORC6 KLK4 TLR10 ORC1 IL27RA ANKRD45 INHBB ALG3 ST6GALNAC5 CXorf58 RYR1 DSC1 PYCR1 UBALD2 NOX4 WDR62 TRIM59 PCDH7 SORT1 HIST1H2BC AK7 NCAPD2 LYPLA1 E2F3 ALDH3A1 GRIN2D RMI2 MYCN FCGR1A MYEF2 GINS2 DRD2 NME1 CHAC2 PCDH19 HTR1D ERCC6L BEND3 RNASEH2A ENKUR SMC4 C3orf67 PAFAH1B3 KIAA1549L CDK5 PITPNM1 ULBP1 CLEC1B PROSER2 SLC25A10 PNP MS4A8 C2orf50 TES MRPL47 MRPL14 MAP4K1 TRPM1 SLC39A4 GINS4 COL13A1 SPC24 MRPL13 SLC7A1 JUP KCTD1 USH1C GPR84 GIPC1 GAPDH MRPL3 STOX1 SYNGR2 PCNA LSM4 DPP3 AHNAK2 RAB3D APLNR EPOP IL12A FUT8 SYNGR3 C20orf204 MRPL15 RTKN BAK1 STRBP RGCC FAM171A2 SLC50A1 SLC39A11 SOWAHA MCUR1 MRPS12 ATP6V0E2 MAP7D2 NRARP GDF9 PIF1 PTX3 UPK2 JPH1 FEN1 TSTA3 PLA2G4F CRIP2 PRC1 DARS2 TMEM19 TRPM2 SIX4 PLD4 E2F1 H2AFX ZNF165 SORD CCDC58 HDGF S100A13 RAB26 CRACR2B LRG1 FAM166B CHRNA5 PRKX TATDN1 CENPL RBM38 ULBP3 TEAD4 MEX3A SDHAF3 CAPN12 FMN1 TONSL ATF5 PRELID1 TPMT RASGEF1C UNC5B DSCC1 AGBL5 COA6 LIN28A PARS2 C5orf22 SLC38A1 NETO2 TRIB3 NDC1 PSMB2 FBP2 SLA2 CFAP74 CACNA1A PYCR3 ZNF93 CHEK1 HIST3H2A TEC PLEKHF2 TIMM17B CCDC160 ZFP57 MUC20 MRPL12 FZD2 DPY30 PPP4C MSH2 CTLA4 MTHFD2 CCNA2 FSD1 ATP6V1F SNX31 NDUFB11 SLC38A4 TMEM223 NAA38 SLC52A2 CENPH MAP2K6 LRGUK NUDT14 PRKCD EXOSC5 DYRK2 RHNO1 SUSD2 TYMS CCDC34 CACNG8 SLC2A12 ETV6 TBCC MOCS3 TIMM23 MRPS15 ATOH7 CCR4 DHFR CYC1 DNMT3B CABP4 YEATS4 LRRIQ3 RCN2 RCC2 SFMBT1 PUF60 CCDC169 COPE PCDHA2 TBC1D31 PFKP ENO1 MGME1 TNFRSF10A FAM81A DERL3 ROMO1 TPI1 CHCHD3 APBA2 HMBS LLGL2 KCNS3 CLPB ANKEF1 PGAM5 EPHA1 FILIP1 MFSD3 FLAD1 TBC1D7 CCNB3 MRPS18A TRIM47 GMIP PNMA3 PDE9A CBX2 ZCRB1 PRIM2 RNF5 NCAPG2 SUV39H2 CSE1L LSM2 BTG3 C5orf34 KIF20B FIBP CDK2AP2 HNRNPAB GPR27 FAM107A PNCK PTGES BATF2 ST3GAL6 EME1 METTL27 RTN3 GPSM3 FNDC11 PTRH1 EIF6 RRAGD SRGAP3 C19orf53 NUDT19 YRDC SHMT2 NOCT PDIA4 BRIP1 ZNHIT1 RUVBL1 SPAG5 GEMIN7 AP1S1 POP7 DNAAF2 LRRC8B DCLRE1B EBP RELB ATP2C2 TMC7 CEBPG CCDC177 CD6 CCT5 NDUFA9 CYCS AGPAT2 ENO3 SNRPC DNPH1 HELZ2 LRRC43 KIF22 NDUFA4L2 ZSCAN16 SLC25A33 SIGLEC5 SAMD1 SAC3D1 DCAF13 BDH1 RAD54B HAUS8 TRMT12 SLC22A18AS C5orf30 YARS2 C1orf74 HSPE1 ADGRE2 VRK1 PAK4 PFDN4 FKBPL UQCC3 EEF1E1 SLC2A6 VIPR1 ACTR3C DDX39A OCA2 FARSA CAPN1 RPP40 IL23A TMEM38A ERI3 B4GALT5 ZFAT CDK16 EDN1 NUDT1 RFC4 TUBG1 ACTL6A PAQR5 NUDT15 SRPK1 RGS16 C1orf127 ZNF365 KLK12 TCF19 PAK1IP1 PRIMA1 RFXANK SNX32 GADD45GIP1 TFPT IPO4 ADAM8 TOMM40 HEY2 LRIG1 ZDHHC9 ZDHHC20 HAPLN3 NIPSNAP1 DNA2 AP1S2 KRAS ZNF440 CLCF1 TRIAP1 YDJC MRPS30 ZDHHC23 ARPC5L BRIX1 WDR77 CENPI FAM189B MROH1 TMEM267 HM13 NDUFAF4 HPRT1 GGCT IER5L MAPK13 RITA1 GMPS IQCD MAGOHB PGP LPAR2 MRPL52 MRPS16 MRPL36 GSTP1 RRP36 TBC1D24 ERGIC2 GFOD1 VPS37D TIMM50 RUVBL2 CNNM4 TTYH3 RFC2 MTX2 ITGAX ZNF239 TOMM34 ANAPC15 IL1RL2 COMMD7 RAVER1 POLE2 DHRS11 MLF2 TMEM54 EFCAB11 PRCC ATAD2 ADA SUV39H1 MRPS34 NEU1 MDK TNFRSF4 ZNF92 TPM4 MCM6 THAP10 RAD23A ZNF787 MRPL33 ACOT7 KLRG1 FAM32A TDP1 CLPTM1L NR2C2AP BABAM1 ALAS1 ADRA2C GMNN DHRS13 FANCD2 PAIP1 C19orf54 INTS5 RPP38 STMN1 MXD3 TOPBP1 MYO6 ABT1 SLC25A39 YES1 PPM1G PUS7 BORA KLHDC9 ESRRA PPP1R16A KEAP1 TMEM63C GLDN LYPLA2 SMARCC1 EHD3 SPDL1 UBE2V2 GATAD2A ATP23 SLCO5A1 POLR2D ARHGEF2 APEX2 CCT3 WDR3 TARBP2 RHEBL1 B3GALT6 ATG101 CHAF1A ZNF593 TLNRD1 SLC7A5 NOL10 S100A3 CDC7 REEP4 |
|  | DOWN | TXNIP RWDD4 MACF1 COBLL1 PDZD2 HMBOX1 GKAP1 EMP3 PLXNA4 IKBKB NFAT5 ZBED5 MBD1 PIGH SEC63 KCTD9 MXI1 RBL2 FAM160B1 THUMPD2 ZNF655 STOM RIC1 ZNF81 EID3 KIF16B FAM160A2 DDX3X DYNC1LI2 RPL3 TBC1D8B ZKSCAN7 TMEM176B RHOBTB1 RCBTB2 TCEAL9 ZNF711 PRKAB2 STAT6 TMEM43 ZCCHC14 TBRG1 SFRP4 USPL1 KIDINS220 ZNF641 ENTPD4 NCBP3 ZCCHC12 MST1 DDHD2 CCNT2 MAP3K3 NPDC1 PCDH9 FAM126A ZBTB4 UBE2G2 CDK20 TXLNG PI4KA HNRNPH3 GGNBP2 PCDH18 TMEM50B PRRX1 ZC3H6 CASD1 TLE2 MARVELD1 KIFC3 PHLPP2 SNAI2 DOCK1 TNFRSF10B CCDC84 COG3 POGZ PRPF3 GNA11 PLSCR4 GNG2 AHNAK MADD EIF3L ERCC6L2 TEP1 OPHN1 KIAA1958 CREBRF ANTXR1 GSE1 RNF146 UHRF2 ZNF407 MAMDC4 RASA1 RAB30 NMNAT3 ERCC5 AKAP9 DDX5 RNF128 ENOSF1 ZCCHC11 MTMR10 FOXP1 GNA12 LYSMD4 ZNF532 NMRK1 MTFR1L SCD5 ZC3H12C SLC26A11 THOC1 B4GAT1 LATS2 RPS6KA2 MORC3 CENPV CWC25 THBD FRY SETD7 ARRDC3 SLC26A6 METTL7A ZNF234 ST13 ZNF266 FERMT2 CBX6 FLNC LAT COLGALT2 AGAP4 GATAD1 GNAZ SERGEF HECA VPS13B HNRNPA1 KATNAL1 MLXIP SLK MSANTD2 BBS7 CDIP1 AKAP13 LIMA1 JOSD1 BTN2A1 NXF1 ZNF493 RIPOR1 CYP4V2 PPWD1 ANKRA2 ARHGAP19 ASB1 ABR SLC7A8 DOCK4 PIM1 MON2 SCN1B IL6ST ZRSR2 MORC4 RAB31 STK11 TRIM4 PCDHB16 CPB1 PDLIM3 ATP9B CTSF SLFN11 ATG2B ADPRM EXOC6B ZNF559 ZNF70 CYBRD1 ZFYVE16 MZF1 CPNE8 ZNF275 OCRL KLHL36 TNRC6A AP1G2 UBXN6 MAP3K4 NEK3 PDE8A ZBTB40 PLBD2 FRMD6 ATP6V0A1 H6PD KAT2B HSPB8 COLCA2 ZZEF1 CLDN15 DENND4A MAP4K5 CGNL1 CLCN5 ARHGEF7 MTMR3 TMEM98 KIZ SMC5 SNX33 PDGFRL ADCY9 RORB TRIM23 HSPA12A CDADC1 MTX3 PALM NEFH NEK1 PLS3 DCUN1D4 FYCO1 HNMT HCFC2 UFSP2 AHI1 GGT5 DDHD1 CHST15 CEP120 CIDEB POLDIP3 ADAMTS3 SETDB2 C5orf56 RAB8B DOCK5 RBPMS2 ZSWIM8 NACC2 FGFR1OP2 PIK3R1 BCO2 HSPB3 GBGT1 TTC8 GNE CRACR2A EPB41L2 MLLT6 CCDC93 XPC EPHX2 DGKH C1RL DIRAS1 MDN1 RALGAPA1 OLFML3 SHPRH PCNX1 LSAMP MPHOSPH8 EHD2 TK2 HERC1 MYSM1 MTHFR MGEA5 RBFOX2 DIP2C FAM13A ABCD4 EID2B SPART SMG6 WDR59 ZNF224 MTURN LONRF1 EVC2 TPM1 C20orf194 NDEL1 PCGF3 MMP16 ERO1B CORO2B EOGT LIFR ING2 KLHL13 DOPEY1 MRC2 DOK6 THRA DENND3 ABLIM1 BRD1 SLC22A17 CCNL1 TMEM8B ATP8B4 PALLD ZEB1 G0S2 SLC9A9 MEF2C CLIC2 FNBP4 TARSL2 PCED1B RXRA IGIP TSPAN4 TNS1 RNASEL ERMARD ICA1L TSC1 DEF8 HYI CLMP YTHDC2 CITED2 ETS2 P2RX7 STMN3 N4BP2L2 BMP2 PAQR7 ADRA2A DNAJC12 ZBTB20 POLI HTRA1 MTERF2 ANGPTL2 ZNF594 TACC1 TGFB1I1 ST3GAL5 DMTF1 CALD1 LIX1L DTX3 PRKAR2B TTBK2 CAV1 MICA MTERF4 ABTB1 CYP27A1 NICN1 C5 PMM1 SSC5D UST SH3PXD2A OGT ID3 JAK2 GULP1 PRKD1 DCHS1 KCNJ8 GCNT7 MICAL1 ARHGAP21 TMEM91 OTUD3 DMD DSCAML1 C11orf95 RHOBTB2 FHL1 SCARB1 SLC16A4 PAXBP1 CLEC2B TOM1L2 PMP22 LRCH2 SYCP2L PRICKLE2 PTGIS CLIP4 CPQ BICC1 EPB41L3 ARHGEF6 NEK9 METAP1D SMARCA2 ANXA6 FBN1 ACCS RUNDC3B GARNL3 SPAG9 NAP1L5 CREBZF LYRM9 TEF IRS1 CAB39L ARMCX1 TPM2 ABLIM3 SLC16A7 RBP1 CAVIN1 NR3C2 GLUL STX2 SECISBP2 LRP1 CAPRIN2 AKR1C2 INTU ARHGEF26 ZHX3 INTS6L FEZ1 FAM13C MITF WNT2B NAP1L3 SH3BP5 TMOD2 EPS8 PRKG1 PAN2 AMIGO2 MAPKBP1 CDH11 SUN2 HEPH ZCCHC24 ARNTL OLFML1 CTNNAL1 KIAA1109 PKD2 SOBP C8orf88 C9orf72 NDN MTUS1 CRY2 WHRN ABCG2 PTPN13 SNAP91 SLC23A2 CHST3 RIC3 SNCA SMPD3 NINL BTAF1 GFRA1 GABBR1 VIM AGAP9 GALNT10 ASH2L C3orf70 ATP8B2 P2RY1 TNNI3K PAPSS2 PHF1 VGLL3 HOXC6 SERP2 KLHDC8B PLCL2 SLC26A4 TCEAL3 TMEM200A TRO SLC25A37 MAF LHFPL6 DYNC1I1 DAPK1 RAPGEF4 CLK1 TRABD2A RBM6 FGF2 TIMP2 EDNRA TFPI ST3GAL4 SESN1 WFS1 TAF1C NFIL3 RGN CASP9 KLHDC1 KANK2 PEX5L MYOM2 ABCA5 WSB1 MICU3 DCLK1 LYVE1 SMAD9 SULF2 TTYH2 AKAP17A FAT4 ZEB2 OMD RUFY3 TBC1D2B NUDT11 FAM198B RGS2 EZH1 SOCS2 MRVI1 RBPMS C1S AKR1C1 RBMS3 COX7A1 PDZRN3 PBX3 GSTM3 NBL1 TSC22D3 ENOX1 EMILIN1 NPHP3 TWIST2 CFH GATM AKAP12 TNS2 ADHFE1 CNRIP1 DCN KLF4 PPP1R12B GFPT2 PARM1 RPRM SMOC2 DST PDE2A COL14A1 VLDLR CPEB1 PLA2R1 PON3 PRSS35 JAM3 PSD3 B3GALT2 CBX7 TCEAL4 ANTXR2 ADGRB3 MRGPRF TBC1D4 LIN7A KLF9 CAPN3 CAVIN2 RHOBTB3 ATP8A1 LRRC17 ARHGAP10 TGFBR3 BAHCC1 WNK4 ZDBF2 SLC7A2 ADH1C CSGALNACT1 PHACTR3 BMPER ITPR1 KCNT2 IGFBP4 HS6ST2 CLDN11 MTUS2 PDE7B KLF2 TSHZ3 MAOA OGN SNCAIP PID1 PABPC5 ZMAT1 YPEL4 TBX3 ZNF331 UBE2Q2L ANKRD29 ROBO3 SLC25A27 MAP3K5 GPAT3 GOLGA8N RAB9B DPT TCF21 PEAR1 ADGRD1 F3 DES COL16A1 MCC RASL11B PDK4 ADAM33 SYNE1 PDGFRA AMHR2 PODN NDNF STARD9 ZNF385B SLC4A3 C21orf62 CXorf57 ADH1B KLHDC8A HAS1 MAMDC2 INHA NRK CMYA5 TDRD10 PLA2G2A C7 HLF SIGLEC11 PEG3 ABCA6 ABCA8 GSTM5 MUM1L1 SCN7A STAR C4BPB WFIKKN2 |
| DEL | UP | LINC01297 LINC01224 PART1 LINC01667 LINC00284 OVAAL LINC00592 LINC00858 LINC01215 LINC01532 LINC01607 LINC00221 LINC00668 LINC00052 LINC00536 HAGLR LINC01136 LINC00867 LINC00665 LINC01558 DLEU1 SAMMSON LINC00996 PCAT6 LINC00240 |
|  | DOWN | FTX LINC00622 MGC16275 C5orf56 LINC00173 GAS1RR LINC00887 LINC00893 |
| DEM | UP | hsa-let-7b-5p hsa-miR-16-5p hsa-miR-23a-3p hsa-let-7f-5p hsa-miR-24-3p hsa-miR-27a-3p hsa-miR-29c-3p hsa-let-7g-5p hsa-let-7i-5p hsa-miR-130a-3p hsa-miR-27b-3p hsa-miR-15b-5p hsa-miR-106b-5p hsa-miR-26b-5p hsa-miR-20a hsa-miR-23b-3p hsa-miR-148a-3p hsa-miR-15a-5p hsa-miR-200c-3p hsa-miR-10b-5p hsa-miR-25-3p hsa-miR-34a-5p hsa-miR-30d-5p hsa-miR-223-3p hsa-miR-1225-5p hsa-miR-342-3p hsa-miR-93-5p hsa-miR-494-3p hsa-miR-30c-5p hsa-miR-331-3p hsa-miR-100-5p hsa-miR-320a-3p hsa-miR-324-3p hsa-miR-20b-5p hsa-miR-497-5p hsa-miR-193a-3p hsa-miR-199a-5p hsa-miR-374a hsa-miR-125b-5p hsa-miR-221-3p hsa-miR-98-5p hsa-miR-574-3p hsa-miR-126-3p hsa-miR-376c hsa-miR-188-5p hsa-miR-148b-3p hsa-miR-136-5p hsa-miR-660-5p hsa-miR-886-3p hsa-miR-155-5p hsa-miR-424-5p hsa-miR-222-3p hsa-miR-381-3p hsa-miR-150-5p hsa-miR-127-3p hsa-miR-132-3p hsa-miR-542-3p hsa-miR-340-3p hsa-miR-340-5p hsa-miR-532-5p hsa-miR-486-5p hsa-miR-202-3p hsa-miR-874-3p hsa-miR-532-3p hsa-miR-362-5p hsa-miR-654-3p hsa-miR-769-5p hsa-miR-505-3p hsa-miR-487b-3p hsa-miR-557 hsa-miR-409-3p hsa-miR-212-3p hsa-miR-337-5p hsa-miR-1228-3p hsa-miR-194-5p hsa-miR-423-5p |
|  | DOWN | hsa-miR-324-5p hsa-miR-551b-5p hsa-miR-194-3p hsa-miR-150-3p hsa-miR-106a-3p hsa-miR-24-2-5p hsa-miR-18a hsa-miR-20b-3p hsa-miR-449b-5p hsa-miR-661 hsa-miR-200a-5p hsa-miR-558 hsa-miR-132-5p hsa-miR-338-5p hsa-miR-374a-3p hsa-miR-193a-5p hsa-miR-185-3p hsa-miR-769-3p hsa-miR-367-3p hsa-miR-15b-3p hsa-miR-136-3p hsa-miR-214-5p hsa-miR-127-5p hsa-miR-222-5p hsa-miR-886-5p hsa-miR-223-5p hsa-miR-10b-3p hsa-miR-331-5p hsa-miR-576-3p hsa-miR-25-5p hsa-miR-376a-5p hsa-miR-16-1-3p hsa-miR-200b-5p hsa-miR-802 hsa-miR-148a-5p hsa-miR-342-5p hsa-miR-106b-3p hsa-miR-34a-3p hsa-let-7g-3p hsa-miR-101-5p hsa-miR-29b-1-5p hsa-miR-195-3p hsa-let-7f-1-3p hsa-miR-92a-1-5p hsa-miR-23a-5p hsa-miR-200c-5p hsa-miR-497-3p hsa-miR-27a-5p hsa-miR-130a-5p hsa-miR-424-3p hsa-miR-23b-5p hsa-miR-30d-3p hsa-miR-27b-5p hsa-miR-100-3p hsa-miR-145-3p hsa-miR-141-5p hsa-miR-125b-1-3p hsa-let-7b-3p |

Table S8 KEGG pathways for genes in ceRNA network

| ID | Description | P value | Gene | Count |
| --- | --- | --- | --- | --- |
| hsa04660 | T cell receptor signaling pathway | 0.017549 | PAK4/VAV3 | 2 |
| hsa05206 | MicroRNAs in cancer | 0.021294 | E2F3/PAK4/ZEB1 | 3 |
| hsa04140 | Autophagy - animal | 0.027357 | RRAGD/STK11 | 2 |
| hsa04068 | FoxO signaling pathway | 0.028963 | SETD7/STK11 | 2 |
| hsa04150 | mTOR signaling pathway | 0.037997 | RRAGD/STK11 | 2 |
