# Supplementary material for: Integrated analysis of lymphocyte infiltration-associated lncRNA for ovarian cancer via TCGA, GTEx and GEO datasets
Source: PeerJ. 2020 May 7;8:e8961. doi: 10.7717/peerj.8961 (PMC7211406; doi:10.7717/peerj.8961)
Supplement: Supplemental Information 8 — Abbreviations: lncRNA, long-non-coding RNA; HGSOC, high-grade serous ovarian cancer; TAM, tumor-associated macrophage; Th, (T helper cell); Tfh, (Follicular helper); T cell, Treg, (regulatory T cell); Cor, R value of Spearman’s correlation. Purity-, no correlation adjusted by purity. Purity+, correlation adjusted by purity. Level of significance: ∗p < 0.01. ∗∗p < 0.001. ∗∗∗p < 0.0001. [file peerj-08-8961-s008.docx]

**Table S9** Correlation analysis between lncRNAs and relate genes and markers of immune cell in HGSOC

|  |  | LINC00858 | | | | |  | FTX | | | | |  | LINC00665 | | | | |
| --- | --- | --- | --- | --- | --- | --- | --- | --- | --- | --- | --- | --- | --- | --- | --- | --- | --- | --- |
|  |  | purity- | |  | purity+ | |  | purity- | |  | purity+ | |  | purity- | |  | purity+ | |
| Description | Gene markers | Cor | P |  | Cor | P |  | Cor | P |  | Cor | P |  | Cor | P |  | Cor | P |
| CD8+ T cell | CD8A | -0.102 | 0.053 |  | -0.095 | 0.071 |  | -0.166 | * |  | -0.095 | 0.07 |  | -0.237 | *** |  | -0.204 | *** |
|  | CD8B | -0.02 | 0.709 |  | 0.088 | 0.092 |  | -0.228 | *** |  | -0.15 | * |  | -0.118 | 0.024 |  | -0.078 | 0.14 |
| T cell (general) | CD3D | -0.15 | * |  | -0.117 | 0.026 |  | -0.166 | * |  | -0.121 | 0.021 |  | -0.224 | *** |  | -0.2 | ** |
|  | CD3E | -0.139 | * |  | -0.114 | 0.03 |  | -0.152 | * |  | -0.089 | 0.092 |  | -0.235 | *** |  | -0.223 | *** |
|  | CD2 | -0.145 | * |  | -0.116 | 0.028 |  | -0.173 | ** |  | -0.133 | 0.011 |  | -0.24 | *** |  | -0.235 | *** |
| B cell | CD19 | -0.008 | 0.878 |  | -0.016 | 0.762 |  | -0.034 | 0.519 |  | -0.018 | 0.738 |  | 0.063 | 0.232 |  | 0.07 | 0.186 |
|  | CD79A | -0.037 | 0.484 |  | -0.053 | 0.317 |  | -0.119 | 0.023 |  | -0.126 | 0.017 |  | -0.092 | 0.082 |  | -0.05 | 0.341 |
| Monocyte | CD86 | -0.16 | * |  | -0.146 | * |  | -0.209 | *** |  | -0.164 | * |  | -0.283 | *** |  | -0.291 | *** |
|  | CSF1R(CD115) | -0.172 | * |  | -0.185 | ** |  | -0.156 | * |  | -0.123 | 0.019 |  | -0.307 | *** |  | -0.229 | *** |
| TAM | CCL2 | -0.185 | ** |  | -0.166 | * |  | -0.137 | * |  | -0.117 | 0.025 |  | -0.191 | ** |  | -0.197 | ** |
|  | CD68 | -0.167 | * |  | -0.182 | ** |  | -0.237 | *** |  | -0.209 | *** |  | -0.302 | *** |  | -0.298 | *** |
|  | IL10 | -0.066 | 0.209 |  | -0.072 | 0.172 |  | -0.145 | * |  | -0.092 | 0.081 |  | -0.246 | *** |  | -0.236 | *** |
| M1 Macrophage | NOS2(INOS) | -0.027 | 0.603 |  | 0.002 | 0.965 |  | -0.099 | 0.059 |  | -0.093 | 0.077 |  | -0.056 | 0.284 |  | -0.08 | 0.13 |
|  | IRF5 | -0.06 | 0.25 |  | -0.059 | 0.262 |  | -0.216 | *** |  | -0.201 | ** |  | -0.122 | 0.02 |  | -0.123 | 0.019 |
|  | PTGS2(COX2) | -0.144 | * |  | -0.044 | 0.4 |  | -0.126 | 0.017 |  | -0.078 | 0.137 |  | -0.184 | ** |  | -0.068 | 0.198 |
| M2 Macrophage | CD163 | -0.147 | * |  | -0.202 | ** |  | -0.221 | *** |  | -0.153 | * |  | -0.283 | *** |  | -0.252 | *** |
|  | VSIG4 | -0.128 | 0.015 |  | -0.136 | * |  | -0.188 | ** |  | -0.126 | 0.016 |  | -0.291 | *** |  | -0.271 | *** |
|  | MS4A4A | -0.157 | * |  | -0.147 | * |  | -0.231 | *** |  | -0.187 | ** |  | -0.288 | *** |  | -0.288 | *** |
| Neutrophils | ITGAM(CD11b) | -0.183 | ** |  | -0.195 | ** |  | -0.137 | * |  | -0.118 | 0.024 |  | -0.31 | *** |  | -0.29 | *** |
|  | CCR7 | -0.087 | 0.098 |  | -0.04 | 0.448 |  | -0.22 | *** |  | -0.185 | ** |  | -0.243 | *** |  | -0.171 | * |
| Natural killer cell | KIR2DL1 | -0.139 | * |  | -0.164 | * |  | -0.059 | 0.263 |  | -0.052 | 0.319 |  | -0.079 | 0.134 |  | -0.079 | 0.131 |
|  | KIR2DL3 | -0.174 | ** |  | -0.084 | 0.109 |  | -0.068 | 0.194 |  | -0.008 | 0.885 |  | -0.18 | ** |  | -0.123 | 0.019 |
|  | KIR2DL4 | -0.175 | ** |  | -0.165 | * |  | -0.112 | 0.033 |  | -0.113 | 0.031 |  | -0.235 | *** |  | -0.216 | *** |
|  | KIR3DL1 | -0.188 | ** |  | -0.181 | ** |  | -0.08 | 0.13 |  | -0.062 | 0.242 |  | -0.157 | * |  | -0.134 | 0.011 |
|  | KIR3DL2 | -0.132 | 0.012 |  | -0.09 | 0.088 |  | -0.047 | 0.37 |  | -0.022 | 0.672 |  | -0.16 | * |  | -0.148 | * |
| Dendritic cell | HLA-DPB1 | -0.138 | * |  | -0.083 | 0.115 |  | -0.103 | 0.051 |  | 0 | 0.997 |  | -0.253 | *** |  | -0.224 | *** |
|  | HLA-DQB1 | -0.108 | 0.039 |  | -0.059 | 0.263 |  | -0.081 | 0.124 |  | 0.056 | 0.284 |  | -0.226 | *** |  | -0.185 | ** |
|  | HLA-DRA | -0.142 | * |  | -0.102 | 0.052 |  | -0.107 | 0.041 |  | -0.02 | 0.71 |  | -0.242 | *** |  | -0.213 | *** |
|  | HLA-DPA1 | -0.146 | * |  | -0.089 | 0.089 |  | -0.129 | 0.014 |  | -0.008 | 0.872 |  | -0.246 | *** |  | -0.234 | *** |
|  | CD1C(BDCA-1) | -0.193 | ** |  | -0.204 | *** |  | -0.036 | 0.491 |  | -0.011 | 0.827 |  | -0.289 | *** |  | -0.268 | *** |
|  | NRP1(BDCA-4) | -0.118 | 0.025 |  | -0.107 | 0.041 |  | -0.173 | ** |  | -0.161 | * |  | -0.133 | 0.011 |  | -0.111 | 0.034 |
|  | ITGAX(CD11c) | -0.167 | * |  | -0.165 | * |  | -0.079 | 0.133 |  | -0.066 | 0.21 |  | -0.283 | *** |  | -0.239 | *** |
| Th1 | TBX21(T-bet) | -0.202 | ** |  | -0.183 | ** |  | -0.2 | ** |  | -0.176 | ** |  | -0.229 | *** |  | -0.206 | *** |
|  | STAT4 | -0.166 | * |  | -0.169 | * |  | -0.147 | * |  | -0.143 | * |  | -0.281 | *** |  | -0.238 | *** |
|  | IFNG(IFN-γ) | -0.076 | 0.15 |  | -0.05 | 0.346 |  | -0.126 | 0.016 |  | -0.103 | 0.051 |  | -0.158 | * |  | -0.128 | 0.014 |
|  | TNF(TNF-α) | -0.086 | 0.103 |  | -0.068 | 0.198 |  | -0.066 | 0.207 |  | -0.085 | 0.105 |  | -0.073 | 0.167 |  | -0.053 | 0.311 |
|  | CXCR3 | -0.13 | 0.013 |  | -0.112 | 0.033 |  | -0.161 | * |  | -0.143 | * |  | -0.249 | *** |  | -0.222 | *** |
|  | CCR5 | -0.169 | * |  | -0.144 | * |  | -0.194 | ** |  | -0.146 | * |  | -0.302 | *** |  | -0.279 | *** |
|  | RUNX3 | -0.013 | 0.801 |  | -0.023 | 0.659 |  | -0.088 | 0.096 |  | -0.106 | 0.044 |  | -0.163 | * |  | -0.145 | * |
|  | EOMES | -0.115 | 0.029 |  | -0.1 | 0.056 |  | -0.155 | * |  | -0.11 | 0.036 |  | -0.235 | *** |  | -0.23 | *** |
| Th2 | GATA3 | -0.086 | 0.101 |  | -0.128 | 0.015 |  | -0.05 | 0.345 |  | -0.061 | 0.244 |  | -0.24 | *** |  | -0.212 | *** |
|  | STAT6 | 0.005 | 0.924 |  | -0.013 | 0.811 |  | 0.161 | * |  | 0.16 | * |  | -0.11 | 0.036 |  | -0.097 | 0.064 |
|  | CCR4 | -0.12 | 0.022 |  | -0.133 | 0.011 |  | -0.131 | 0.012 |  | -0.114 | 0.03 |  | -0.259 | *** |  | -0.245 | *** |
|  | CCR8 | -0.049 | 0.355 |  | -0.061 | 0.248 |  | -0.098 | 0.063 |  | -0.089 | 0.091 |  | -0.203 | ** |  | -0.166 | * |
|  | IRF4 | -0.126 | 0.016 |  | -0.024 | 0.648 |  | -0.153 | * |  | -0.121 | 0.022 |  | -0.21 | *** |  | -0.102 | 0.051 |
| Tfh | BCL6 | -0.196 | ** |  | -0.172 | * |  | 0.153 | * |  | 0.138 | * |  | -0.191 | ** |  | -0.178 | ** |
|  | IL6 | -0.085 | 0.104 |  | -0.028 | 0.589 |  | -0.152 | * |  | -0.171 | * |  | -0.174 | ** |  | -0.142 | * |
|  | IL27 | -0.103 | 0.05 |  | -0.076 | 0.149 |  | -0.186 | ** |  | -0.169 | * |  | -0.211 | *** |  | -0.214 | *** |
|  | IL12A | 0.008 | 0.875 |  | -0.036 | 0.489 |  | -0.073 | 0.163 |  | -0.049 | 0.347 |  | -0.116 | 0.027 |  | -0.091 | 0.085 |
|  | IL12B | -0.047 | 0.371 |  | -0.016 | 0.765 |  | -0.123 | 0.019 |  | -0.117 | 0.026 |  | -0.147 | * |  | -0.123 | 0.019 |
|  | IL10 | -0.066 | 0.209 |  | -0.072 | 0.172 |  | -0.145 | * |  | -0.092 | 0.081 |  | -0.246 | *** |  | -0.236 | *** |
|  | CXCR5 | -0.085 | 0.106 |  | -0.077 | 0.145 |  | 0.003 | 0.949 |  | 0.007 | 0.9 |  | -0.031 | 0.56 |  | -0.03 | 0.571 |
|  | CD40LG(CD40L) | -0.144 | * |  | -0.14 | * |  | -0.098 | 0.061 |  | -0.077 | 0.142 |  | -0.174 | ** |  | -0.124 | 0.018 |
|  | BCL6 | -0.196 | ** |  | -0.172 | * |  | 0.153 | * |  | 0.138 | * |  | -0.191 | ** |  | -0.178 | ** |
|  | STAT3 | -0.072 | 0.169 |  | -0.067 | 0.203 |  | -0.219 | *** |  | -0.227 | *** |  | -0.251 | *** |  | -0.236 | *** |
|  | MAF(c-MAF) | -0.15 | * |  | -0.154 | * |  | -0.101 | 0.055 |  | -0.087 | 0.096 |  | -0.252 | *** |  | -0.235 | *** |
|  | HNF1A(TCF1) | 0.134 | 0.011 |  | 0.106 | 0.044 |  | 0.026 | 0.618 |  | 0.021 | 0.69 |  | 0.144 | * |  | 0.165 | * |
|  | IRF4 | -0.126 | 0.016 |  | -0.024 | 0.648 |  | -0.153 | * |  | -0.121 | 0.022 |  | -0.21 | *** |  | -0.102 | 0.051 |
|  | LEF1 | 0.141 | * |  | 0.11 | 0.036 |  | -0.009 | 0.86 |  | 0.004 | 0.935 |  | 0.178 | ** |  | 0.159 | * |
|  | NFATC1(NFAT2) | -0.229 | *** |  | -0.199 | ** |  | -0.004 | 0.936 |  | 0.057 | 0.281 |  | -0.242 | *** |  | -0.221 | *** |
| Th17 | STAT3 | -0.072 | 0.169 |  | -0.067 | 0.203 |  | -0.219 | *** |  | -0.227 | *** |  | -0.251 | *** |  | -0.236 | *** |
|  | IL6 | -0.085 | 0.104 |  | -0.028 | 0.589 |  | -0.152 | * |  | -0.171 | * |  | -0.174 | ** |  | -0.142 | * |
|  | IL23A | -0.015 | 0.783 |  | -0.008 | 0.875 |  | 0.066 | 0.211 |  | 0.079 | 0.131 |  | -0.005 | 0.921 |  | -0.019 | 0.712 |
|  | TGFB1(TGFβ) | -0.119 | 0.024 |  | -0.133 | 0.012 |  | -0.248 | *** |  | -0.229 | *** |  | -0.112 | 0.033 |  | -0.091 | 0.084 |
|  | CCR6 | -0.13 | 0.013 |  | -0.134 | 0.01 |  | -0.031 | 0.562 |  | -0.05 | 0.344 |  | -0.222 | *** |  | -0.207 | *** |
|  | RORA(RORα) | -0.01 | 0.843 |  | 0.041 | 0.44 |  | 0.103 | 0.05 |  | 0.129 | 0.014 |  | -0.15 | * |  | -0.144 | * |
| tTreg | FOXP3 | -0.007 | 0.887 |  | 0.002 | 0.964 |  | -0.219 | *** |  | -0.192 | ** |  | -0.193 | ** |  | -0.19 | ** |
|  | TGFB1(TGFβ) | -0.119 | 0.024 |  | -0.133 | 0.012 |  | -0.248 | *** |  | -0.229 | *** |  | -0.112 | 0.033 |  | -0.091 | 0.084 |
|  | IL10 | -0.066 | 0.209 |  | -0.072 | 0.172 |  | -0.145 | * |  | -0.092 | 0.081 |  | -0.246 | *** |  | -0.236 | *** |
|  | TBX21(T-bet) | -0.202 | ** |  | -0.183 | ** |  | -0.2 | ** |  | -0.176 | ** |  | -0.229 | *** |  | -0.206 | *** |
| T cell exhaustion | PDCD1(PD-1) | -0.063 | 0.229 |  | -0.034 | 0.522 |  | -0.219 | *** |  | -0.193 | ** |  | -0.169 | * |  | -0.173 | ** |
|  | CTLA4 | -0.161 | * |  | -0.11 | 0.037 |  | -0.216 | *** |  | -0.19 | ** |  | -0.267 | *** |  | -0.234 | *** |
|  | LAG3 | -0.106 | 0.043 |  | -0.096 | 0.066 |  | -0.117 | 0.025 |  | -0.135 | * |  | -0.091 | 0.083 |  | -0.091 | 0.083 |
|  | HAVCR2(TIM-3) | -0.147 | * |  | -0.134 | 0.011 |  | -0.195 | ** |  | -0.128 | * |  | -0.298 | *** |  | -0.297 | *** |
|  | GZMB | -0.157 | * |  | -0.085 | 0.107 |  | -0.163 | * |  | -0.108 | 0.04 |  | -0.177 | ** |  | -0.169 | * |

**Abbreviations**: lncRNA, long-non-coding RNA. HGSOC, high-grade serous ovarian cancer. TAM, tumor-associated macrophage. Th (T helper cell), Tfh (Follicular helper), T cell, Treg (regulatory T cell). Cor, R value of Spearman’s correlation. Purity-, no correlation adjusted by purity. Purity+, correlation adjusted by purity.

Level of significance: **p < 0.01. **p < 0.001. ***p < 0.0001.*
